# Vaccinosis and Its Cure by Thuja, with Remarks on Homœoprophylaxis

**Published:** 1884-08

**Authors:** 


### BOOK NOTICE.

VACCINOSIS AND ITS CURE BY THUJA, WITH REMARKS ON HOMŒOPROPHYLAXIS. By J. Compton Burnett, M. D. Pp. 126, demi-octavo. Price, seventy-five cents. London: The Homœopathic Publishing Co., 12 Warwick Lane, E. C. New York and Philadelphia: F. E. Boericke. 1884.

Dr. Burnett has written a number of practical and highly interesting essays, upon which we commented some time since. The subject of the present essay is by no means less important or less practical than any of its predecessors.

Vaccination is performed so extensively that any knowledge of the diseases following it must be of prime importance Nor is a knowledge of prophylactic medicine of less importance; in fact, the field of homœoprophylaxis has scarcely been entered upon, much less cultivated to any extent. The law of the similars rightly studied and used is undoubtedly the best guide

1. For curing all curable diseases.
2. For palliating the incurable.
3. For Prophylaxis, scientific preventive medicine.

The purpose of the volume we are now considering is to show: "First, that there exists a diseased state of the constitution which is engendered by the vaccinal virus (the so-called lymph), which state he (the author) proposes to call VACCINOSIS, or the *vaccinal state*, and secondly, that there exists also in nature a notable remedy for Vaccinosis, viz.: The *Thuja Occidentalis*, and thirdly, that *Thuja* is a remedy of Vaccinosis by reason of its homœopathicity thereto, and fourthly, that the law of the similars also applies to the prevention of disease.

The relation between Vaccinosis and Thuja is clearly shown by Dr. Burnett in a series of highly interesting cases cured by that potent remedy. In the matter of homœoprophylaxis, our author does not show such strong testimony as before in Vaccinosis, for this latter field is of a different nature and less cultivated. Nevertheless, we cannot fail to assent to his propositions, especially the following quotation: "It seems to me that the requirements of the age is to *systematize* the prevention of disease according to the law of the similars AND IN THE DYNAMIC DOSE. Clearly the dynamic dose is ESSENTIAL, or at any rate the very small dose, for otherwise the homœoprophylactic aggravation would be a serious detriment in every way."

Homœopathic vaccination, that is, the vaccine matter prepared as an homœopathic remedy and given by the mouth in dynamic dose, is recommended as the homœoprophylactic.

We would strongly urge our readers to carefully read this interesting volume.

